# GFAPδ: A Promising Biomarker and Therapeutic Target in Glioblastoma

**DOI:** 10.3389/fonc.2022.859247

**Published:** 2022-03-18

**Authors:** Roxana Radu, George E. D. Petrescu, Radu M. Gorgan, Felix M. Brehar

**Affiliations:** ^1^Department of Neurosurgery, Carol Davila University of Medicine and Pharmacy, Bucharest, Romania; ^2^Department of Neurosurgery, Bagdasar-Arseni Clinical Emergency Hospital, Bucharest, Romania

**Keywords:** GFAP, glioblastoma, GFAPδ/α ratio, invasiveness, subventricular zone

## Abstract

GFAPδ, the delta isoform of the glial fibrillary acidic protein, is mainly expressed in the subventricular zone of the brain, together with other neural stem cell markers like nestin. The authors of this paper were among the first that described in detail the expression of GFAPδ and its correlation with malignancy and invasiveness in cerebral astrocytoma. Later, several papers confirmed these findings, showing that the alternative splice variant GFAPδ is overexpressed in glioblastoma (CNS WHO grade 4) compared with lower grade gliomas. Other studies suggested that a high GFAPδ/α ratio is associated with a more malignant and invasive behavior of glioma cells. Moreover, the changing of GFAPδ/α ratio affects the expression of high-malignant genes. It is now suggested that discriminating between predominant GFAP isoforms, GFAPδ or GFAPα, is useful for assessing the malignancy state of astrocytoma, and may even contribute to the classification of gliomas. Therefore, the purpose of this paper is to review the literature with emphasize on the role of GFAPδ as a potential biomarker, and as a possible therapeutic target in glioblastoma.

## Introduction

Glial fibrillar acid protein (GFAP) is a type III intermediate filament protein (IF) found in the cytoskeleton of central nervous system’s (CNS) glial cells (1). GFAP molecule contains 432 aminoacids and has head and tail domains flanking a central α-helical rod domain. Interestingly, during the evolution, more than 90% of the amino acid sequence is conserved among human, mouse, and rat (2). GFAP molecules, after posttranslational modification (mainly phosphorylation and citrullination), start assembling in a multistep process like other type III intermediate filament proteins (3). This process is initiated with monomers binding in a parallel fashion to form dimers, then tetramers are formed by antiparallel association of dimers, followed by lateral bindings that produce octamers, oligomers, and the final filament structures (4).

GFAP is largely expressed in the astrocytes of the central nervous system, but it can also be found in nonmyelinating Schwann cells and enteric glia. GFAP is expressed not only in normal brain tissue, but also in brain tumors like astrocytoma, where it is one of the most important markers for astrocyte lineage. GFAP is also expressed in other tumors like ependymoma (5), pleomorphic xanthoastrocytoma (6), and in other unexpected sites such as myoepithelial tissue and salivary gland tumors (7).

Since its first report by Eng et al. in 1969 (1, 8), 6 isoforms have been described, from human and rodent sources, with splice variants at both 5’ and 3’ ends (**Table 1**) (11–15, 22, 23).

**Table 1:** Research on GFAP related to the invasiveness of cerebral gliomas.

Research Highlight	Reference	Country	Summary
Discovery – canonical isoform α	Eng et al., 1971 (9)	U.S.A.	The isolation of an acidic protein as a major component of human brain tissue with severe fibrillary gliosis, that would later be called GFAP.
GFAP term into common use	Uyeda et al., 1972 (10)	U.S.A.	Immunological study - normal human brain and astrocytoma cross-react with anti-GFA antibodies.
GFAP β	Feinstein et al., 1992 (11)	U.S.A.	Description of a new splice variant, which initiates upstream to the major start site and is found predominantly in Schwann cells.
GFAP γ	Zelenika et al., 1995 (12)	France	New splice transcript, which contains a part of the intron 1, is expressed in mouse bone marrow and spleen as well as in human and mouse central nervous system.
GFAPδ	Condorelli et al., 1999 (13)	Italy	Novel transcript with exon 7a, which replaces the exons 8 and 9 from GFAPα. It was isolated from rat hippocampus.
GFAPƐ	Nielsen et al., 2002 (14)	Denmark	This splice variant is characterized by a new C-terminal protein sequence, and has the ability to specifically bind presenilin proteins in yeast and *in vitro*.
GFAP κ	Blechingberg et al., 2007 (15)	Denmark	Latest isoform produced by alternative splicing and polyadenylation of the 3’-region of the human GFAP pre-mRNA.
GFAPδ expression in subventricular zone	Roelofs et al., 2005 (16)	Netherlands	Neural stem cells in the adult human brain actively splice GFAP-delta transcripts.
Neural stem cells and the origin of gliomas	Sanai et al., 2005 (17)	U.S.A.	The transformation of SVZ astrocytes with stem features is the basis of gliomagenesis.
GFAPδ immunostaining in cerebral astrocytomas	Brehar et al., 2015 (18)	Romania	GFAPδ and nestin-positive cells in cerebral astrocytomas correlates with tumor invasiveness assessed by preoperative neuroimaging investigations.
GFAPδ/α ratio and expression of malignant genes	Stassen et al., 2017 (19) Moeton et al, 2014 (20)	Netherlands	DUSP4 expression in glioma correlates with the GFAPδ/α ratio, and high expression is associated with a worse prognosis. LAMA1 associated with gliomas invasion was increased in cells with a high GFAPδ expression compared to GFAPα.
GFAPδ/α ratio and glioma invasiveness	Uceda-Castro et al., 2022 (21)	Netherlands	High-grade gliomas are associated with GFAPα down-regulation and and increased GFAPδ.

The most abundant GFAP isoform in glial cells is GFAPα, which is the 432 amino acid protein homomerically assembled. The next GFAP isoforms discovered, GFAPβ and GFAPγ, are different from the main isoform by RNA start sites, with GFAPβ mRNA being upstream of that of GFAPα (11, 24) and GFAPγ beginning transcription at 130 nucleotides from the end of GFAPα intron 1 (12). GFAPβ and GFAPγ splice variants carry downstream of their transcription start sites the GFAPα exons. Among cytoskeleton intermediate filaments, alternative splicing is a well-described process in GFAP and synemin, producing additional isoforms (25). Other isoforms of interest produced by alternative splicing are GFAPδ and the latest described, GFAP kappa (**Table 1**) (15).

## GFAPδ – Molecular Structure and Expression

In 1999, Condorelli et al. discovered a new transcript named GFAPδ, which was isolated from the rat hippocampus (**Table 1**) (13). GFAPδ transcript contained a previously undetected exon, exon 7a, which replaces the exons 8 and 9 from GFAPα. The result is a distinct C-terminal tail domain of GFAPδ compared to GFAPα sequence. Exon 7a, which is present in all mammals, including humans, is unique by its splice acceptor site and polyadenylation signal (26). From a functional viewpoint, the difference in the C-terminal tail domain is crucial. Therefore, GFAPδ by itself can aggregate and prevent normal filament assembly if its concentration (induced by transfection of astrocytic cell line) reaches a threshold concentration (10-30% of total GFAP) (27, 28).

**Figure 1:**
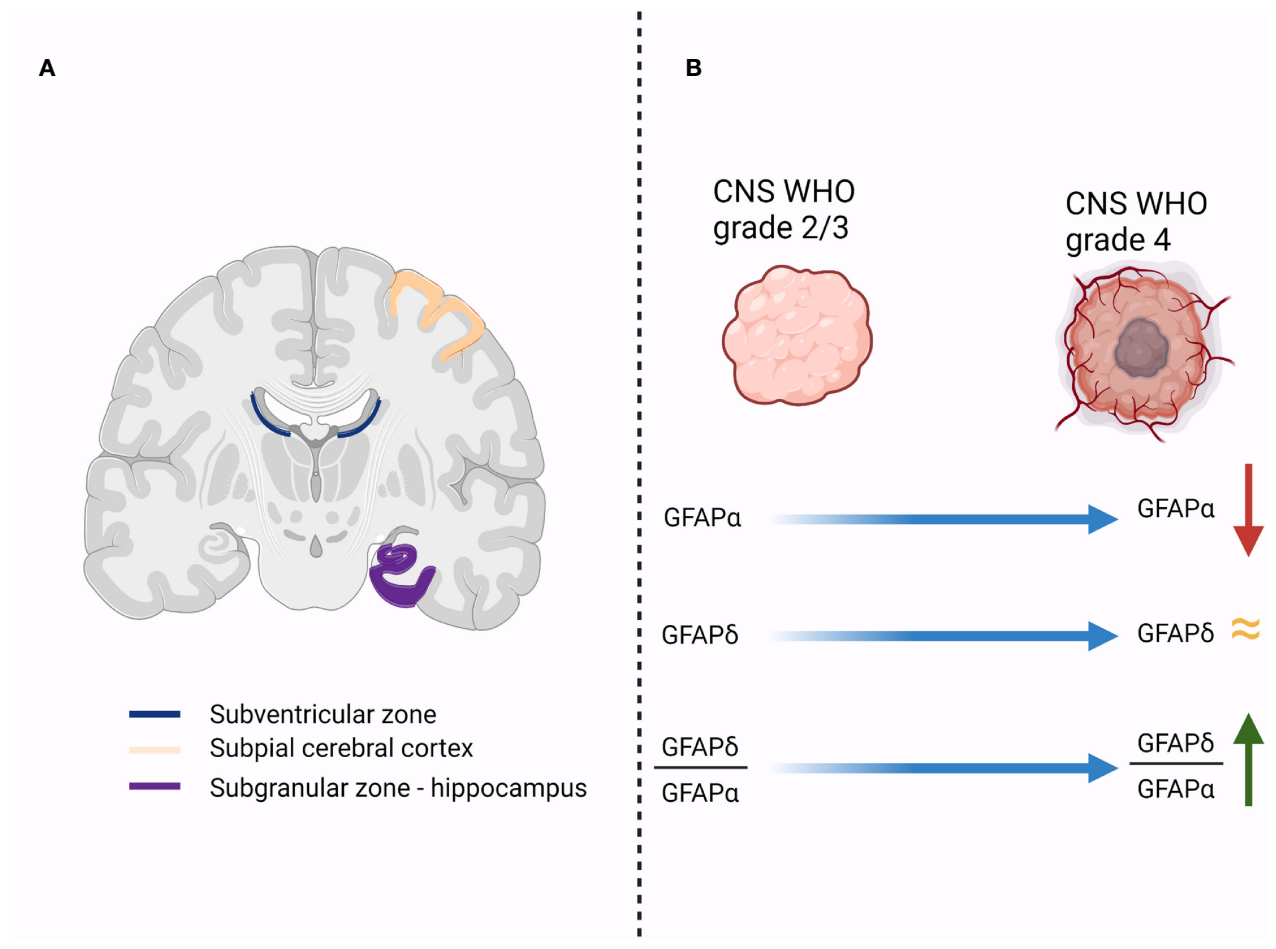
**(A)**. Illustrates the regions in the human brain that have a high expression of GFAPδ-positive cells (subventricular zone, subpial cerebral cortex and subgranular zone of the hippocampus); **(B)**. Initial studies showed that the expression of GFAPα decreases in higher grade gliomas, while the level of GFAPδ remains relatively the same. Therefore, the ratio between GFAPδ and GFAPα expression increases in higher-grade tumors, and it is associated with a more malignant profile.

The subventricular zone (SVZ) is a distinct region of the brain with specific features. One of the most important characteristics of this area is the presence of particular cell populations with stem-like properties. Numerous studies have identified a subpopulation of astrocytes as the multipotent neural stem cells (NSCs) of the adult mammalian brain (29–33). Interestingly, Roelofs and colleagues found that within all the areas that were tested from human postmortem brain specimens, the largest localization of GFAPδ immunopositive astrocytes was in the subependymal layer of each lateral ventricle (**Figure 1A**) (16). These astrocytes have a particular phenotype and form a ribbon of cells along the lateral ventricles (16). Even though the authors found that the population of GFAPδ -positive cells in the SVZ is considerably higher than the number of NSC in this area [approximated by Morshead et al. at 0.2-0.4% NSC (34)], they considered that a certain subgroup of GFAPδ -positive SVZ astrocytes represent the multipotent NSC (16).

Another study, published by van den Berge and colleagues, demonstrated that GFAPδ expressing cells were found not only in the SVZ but also in the rostral migratory stream (RMS) on the way to the olfactory bulb (35). Nestin, a marker for NSCs, proliferating cell nuclear antigen (PCNA) and Mcm2, which are cell proliferation markers are expressed simultaneously with GFAPδ in these cells (35). The authors support this hypothesis with evidence that GFAPδ expressing cells in the SVZ resemble immature astrocytes with neural stem cells behavior. Furthermore, vimentin, a marker for immature astrocytes and the general astrocyte marker GFAPα are co-expressed in these cells (8). Notably, GFAPδ-positive cells lacked expression of late markers of astrocyte development, such as glutamine synthetase (GS) and S100B. More important, to support their assertion is the evidence of co-expression, in GFAPδ-positive cells of the transcription factor Sox2, important for the maintenance of adult neurogenesis (36). Based on these results, GFAPδ acts as a marker of NSCs in the SVZ.

Sanai and colleagues raised the hypothesis that SVZ is probably the origin of cerebral gliomas (17). The transformation of these astrocytes with stem features, which occurs in the SVZ, followed by outward migration, could be the origin of astrocytomas (17). As these astrocytes (which reside in the SVZ) express GFAPδ, a reasonable assumption is that cerebral astrocytoma may retain the molecular signature and express GFAPδ. Our previous study confirmed this hypothesis and has demonstrated a statistically significant correlation between the grade of GFAPδ immunostaining and the grade of nestin immunostaining in cerebral astrocytoma (**Table 1**) (37). Moreover, a statistically significant correlation was found between the neuroimaging invasiveness of cerebral astrocytoma and GFAPδ immunostaining grade (18).

## GFAPδ as a Marker of Invasiveness in Malignant Astrocytoma

Cerebral astrocytoma is the most common primary cerebral tumor, with an incidence slightly higher in the male population and is commonly encountered in adult age (38). There are four-grade astrocytomas, with CNS WHO grade 4 being the most malignant type with a median survival despite combined treatment (radical surgical resection followed by radiotherapy and chemotherapy) of approximately 15 months (39). The new WHO 2021 classification system includes several biomarkers to classify cerebral gliomas and to better predict the malignant behavior of these tumors (40). Adult type diffuse gliomas are therefore classified as isocitrate dehydrogenase (IDH)-mutant astrocytoma (graded CNS WHO 2/3/4), IDH mutant and 1p/19q codeleted oligodendroglioma (graded CNS WHO 2/3) and glioblastoma (GBM) IDH-wildtype (graded CNS WHO 4). Grading is based on natural history and invasiveness (40).

Invasion is one of the most important pathological features, which precludes total resection and favors an early tumor recurrence. Certain glioblastomas (CNS WHO grade 4 astrocytomas) have an unusual short clinical course to recurrence after radical resection followed by radiotherapy and chemotherapy and display a highly invasive re-growth pattern with tumor infiltration in the contralateral cerebral hemisphere or satellite tumors developed in distant locations from the original tumor site.

Several markers have been used to determine the prognostic of glioblastoma patients. The evaluation of the Ki-67 labelling index has been described for glioblastoma, but the existing data are controversial whether there is (41, 42) or not (43–45) a benefit on survival. The methylation test for *MGMT* (O6-methylguanine DNA methyltransferase) promoter is one of the most commonly used predictive markers (46) and while numerous studies have reported that the hypermethylation of *MGMT* promoter is associated with increased overall survival (OS) and progression-free survival (PFS) (47), other authors report no difference between the survival of patients with *MGMT* methylation and those without (48). Moreover, Poon et al. showed that the methylation status of the *MGMT* promoter influenced only the survival of patients that did not complete the temozolomide regimen, having a limited impact on the survival of patients that completed the regimen (49).

Regarding the role of GFAP as a biomarker, Ahmadipour et al. demonstrated by using immunohistochemical staining of paraffin-embedded glioblastoma samples that a GFAP value ≥75% is associated with worse survival, independent of the *MGMT* promoter methylation status or extent of resection (50). Another study by Sommerlath et al. assessed the differences between long and short-term survivors regarding the GFAP expression, *MGMT* status and Ki-67 index (51). A decreased Ki-67 index was observed in patients with increased survival, but the difference was significant only when compared with one of the two short-term survivor groups that were included in the study (51). *MGMT* promoter hypermethylation and GFAP-positive tumors were significantly associated with increased OS when compared to both short-term survivor groups and patients with GFAP-positive tumors had a longer survival independent of the *MGMT* promoter status (51). Considering these contradictory findings regarding the role of the overall expression of GFAP as a prognostic factor for glioblastoma patients, it is necessary to evaluate the expression of the GFAP isoforms.

Several studies highlighted the expression of high levels of GFAPδ in neurogenic stem cells (16, 35, 52) and in high-grade astrocytomas compared to lower-grade ones (37, 53, 54).

With the objective to accurately distinguish the differentiation state of astrocytomas, it will be necessary to assess the predominant GFAP isoform expression, either GFAPα or GFAPδ (55). Accordingly, the GFAPδ/α ratio is increased in grade IV astrocytoma (**Figure 1B**) (19).

Moeton et al. proved that increased GFAPδ expression changes the interaction of astrocytoma cells with the microenvironment, with significantly decreased motility by down-regulation of plectin, a protein involved in the filaments network and over-expression of the extracellular matrix component laminin (20).

Uceda-Castro et al., with the use of ex vivo brain slice invasion model and intravital imaging, showed different migratory dynamics of glioma cells depending on the GFAPδ and GFAPα expression levels. High-grade gliomas are associated with alternative splicing in GFAP expression, as GFAPα is downregulated while GFAPδ has an increased dominance in these tumors (**Table 1**) (21).

Also, GFAPδ showed to be a reliable marker for spinal cord astrocytoma diagnosis, with GFAPδ immunoreactivity being significantly correlated with spinal cord astrocytoma grade (56).

Kanski et al. demonstrated that inhibition of histone deacetylases (HDACs) reduces GFAP expression in astrocytoma cells and the ratio between GFAPδ and canonical isoform GFAPα increases in favor of GFAPδ (57). Histone alteration plays an essential role in glioblastoma genesis, progression and treatment resistance and depends on two types of enzymes, histone acetyltransferases (HATs) and HDACs. To maintain this balance, HDAC inhibitors (HDACis) are identified as novel agents for cancer therapy (58).

### GFAPα/GFAPδ Ratio and the Malignant Profile of Cerebral Astrocytoma

Further studies focused on the expression of GFAP isoforms in cerebral astrocytomas showed that, while GFAPα expression is significantly lower in grade IV astrocytomas compared to grade II and grade III astrocytomas, the expression of the alternative splice variant GFAPδ tends to be maintained between astrocytoma grades (19). The result is an increase in GFAPδ expression compared to GFAPα, translated by higher GFAPδ/α ratio in grade IV astrocytoma compared to lower grade (**Figure 1B**) (19). More important, a higher GFAPδ/α ratio is not only an epiphenomenon associated with malignant profile of cerebral astrocytoma (19). Stassen and colleagues demonstrated that GFAPδ/α ratio regulates high-malignant genes and many of those genes are involved in the regulation of important biological process like the mitotic cell cycle, regulation of cell proliferation and regulation of phosphorylation (19). Therefore, the conclusion of the authors was that while searching for novel therapeutic targets for cerebral astrocytomas, modulating GFAP isoforms expression and selectively splicing should be considered (19).

Interestingly, a higher GFAPδ/α ratio induces not only changes in the genetic expression that regulates the biological process of the astrocytic cells, but also it activates genes involved in the interaction between glioma cells and the extracellular matrix (ECM) (59). One of the key molecules activated by an increased GFAPδ/α ratio *in vitro* is the dual-specificity phosphatase 4 (DUSP4), also called MAPK phosphatase 2 (19). In glioma patients, DUSP4 expression correlates with the GFAPδ/α ratio, and high expression is associated with worse prognosis (**Table 1**). This phosphatase plays a key role in MAPK-signaling pathway, which in turn regulates various tumor malignancy–related processes. In gliomas, mutations in the MAPK pathway and constitutive activation of the DUSP4 that target ERK and Janus kinase (JNK) are common (60–62). Moreover, DUSP4 activity influences key biological process dysregulated in gliomas like cell migration (63), invasion (64), proliferation (65), ECM degradation (66), and chemotherapy-induced cytotoxicity (67–69).

Another important gene, *LAMA1*, which encodes the laminin alpha1 chain of the ECM molecule laminin-111, was significantly increased in cells with a high GFAPδ/α ratio (19, 20). Other previous experiments demonstrated that GFAPδ/α ratio influenced the expression of a downstream effector of laminin-signaling activity, metalloproteinase 2 (19, 70). This is a well-studied metalloproteinase involved in cell invasion (71, 72) and is associated with glioma malignancy (73). Therefore, concerning cell–ECM interaction pathways changed by DUSP4 status, van Bodegraven and colleagues show that a high GFAPδ/α ratio enables glioma cells to have a greater invasiveness capability in the brain (**Table 1**) (55).

## Future Directions and Conclusion

The poor prognosis of glioblastoma is primarily related to the local invasiveness and the tendency to relapse due to the radio- and chemotherapy resistance after surgical resection.

A recent meta-analysis also showed that GFAP levels measured from serum can be used to identify glioblastoma, but further studies are needed since currently the sensitivity of this method is still poor (74). Therefore, the assessment of GFAP in biofluids has a limited role.

However, when performing a biopsy procedure or a surgical resection of a glioblastoma, the high expression of GFAPδ, an alternative splice variant of GFAP, could predict the invasiveness and the increased risk for tumor recurrence. Therefore, it would be useful to regularly assess the immunohistochemical expression of GFAPδ (together with other glioblastoma markers) and patients with increased expression of GFAPδ in the glioblastoma samples from the initial surgery should be closely monitored after surgery. These patients should be stratified as high risk of early recurrence and should be closely followed-up by regular neuroimaging investigations.

Since a high GFAPδ/α ratio is associated with the expression of high-malignant genes and migratory dynamics of glioma cells, novel therapies should focus on balancing the ratio between GFAPα and GFAPδ to decrease the motility and invasiveness of malignant glioma cells. The regulation of histone acetylation has an essential role in glioblastoma and could be a promising target by reducing GFAP total expression. To date, new HDAC inhibitors are under study.

## Author Contributions

FB conceptualized the paper. FB and GP made the figure. All authors performed the literature search, wrote the manuscript, and reviewed and approved the final form of the manuscript.

## Conflict of Interest

The authors declare that the research was conducted in the absence of any commercial or financial relationships that could be construed as a potential conflict of interest.

## Publisher’s Note

All claims expressed in this article are solely those of the authors and do not necessarily represent those of their affiliated organizations, or those of the publisher, the editors and the reviewers. Any product that may be evaluated in this article, or claim that may be made by its manufacturer, is not guaranteed or endorsed by the publisher.
